# Screening Perspectives: The Role of Colorectal Cancer Awareness in Shaping Attitudes Toward Colonoscopy in Palestine

**DOI:** 10.1200/GO.23.00470

**Published:** 2024-02-22

**Authors:** Mohamedraed Elshami, Maram Albandak, Mohammed Alser, Ibrahim Al-Slaibi, Mohammed Ayyad, Mohammad F. Dwikat, Shoruq A. Naji, Balqees M. Mohamad, Wejdan S. Isleem, Adela Shurrab, Bashar Yaghi, Yahya Ayyash Qabaja, Fatma K. Hamdan, Raneen R. Sweity, Remah T. Jneed, Khayria A. Assaf, Mohammed M. Hmaid, Iyas I. Awwad, Belal K. Alhabil, Marah N. Alarda, Amani S. Alsattari, Moumen S. Aboyousef, Omar A. Aljbour, Rinad AlSharif, Christy T. Giacaman, Ali Y. Alnaga, Ranin M. Abu Nemer, Nada M. Almadhoun, Sondos M. Skaik, Shurouq I. Albarqi, Nasser Abu-El-Noor, Bettina Bottcher

**Affiliations:** ^1^ Division of Surgical Oncology, Department of Surgery, University Hospitals Cleveland Medical Center, Cleveland, OH; ^2^ Ministry of Health, Gaza, Palestine; ^3^ Faculty of Medicine, Al-Quds University, Jerusalem, Palestine; ^4^ The United Nations Relief and Works Agency for Palestine Refugees in the Near East (UNRWA), Gaza, Palestine; ^5^ Almakassed Hospital, Jerusalem, Palestine; ^6^ Faculty of Medicine, An-Najah National University, Nablus, Palestine; ^7^ Faculty of Pharmacy, Al-Azhar University of Gaza, Gaza, Palestine; ^8^ Doctors Without Borders (Médecins Sans Frontières), Hebron, Palestine; ^9^ Faculty of Medicine, Islamic University of Gaza, Gaza, Palestine; ^10^ Palestine Medical Complex, Khanyounis, Palestine; ^11^ Faculty of Dentistry, Arab American University, Jenin, Palestine; ^12^ Augusta Victoria Hospital, Jerusalem, Palestine; ^13^ Faculty of Allied Medical Sciences, Arab American University, Jenin, Palestine; ^14^ Faculty of Medicine, Al-Azhar University, Gaza, Palestine; ^15^ Faculty of Nursing, Islamic University of Gaza, Gaza, Palestine

## Abstract

**PURPOSE:**

To assess colorectal cancer (CRC) awareness and its influence on attitudes toward colonoscopy in Palestine.

**MATERIALS AND METHODS:**

Convenience sampling was used to recruit Palestinian adults from hospitals, primary health care centers, and public spaces across 11 governorates. To evaluate the awareness of CRC signs/symptoms, risk factors, and mythical causes, the Bowel Cancer Awareness Measure and Cancer Awareness Measure-Mythical Causes Scale were used after translation into Arabic. For each correctly recognized item, one point was given. The total awareness score of each domain was calculated and categorized into tertiles; the top tertile was considered high awareness, and the other two tertiles were considered low awareness.

**RESULTS:**

A total of 4,623 questionnaires were included. Only 1,849 participants (40.0%) exhibited high awareness of CRC signs/symptoms. High awareness of CRC symptoms was associated with higher likelihood of showing positive attitudes toward colonoscopy (odds ratio [OR], 1.21 [95% CI, 1.07 to 1.37]). A total of 1,840 participants (38.9%) demonstrated high awareness of CRC risk factors. Participants with high awareness of CRC risk factors were more likely to display positive attitudes toward colonoscopy (OR, 1.20 [95% CI, 1.07 to 1.37]). Only 219 participants (4.7%) demonstrated high awareness of CRC causation myths. There was no association between awareness of CRC causation myths and positive attitudes toward colonoscopy.

**CONCLUSION:**

Awareness of CRC was poor with less than half of the study participants demonstrating high awareness of CRC signs/symptoms and risk factors, and a minority (<5%) displaying high awareness of CRC causation myths. High awareness of CRC signs/symptoms and risk factors was associated with greater likelihood of demonstrating positive attitudes toward colonoscopy. Educational initiatives are needed to address knowledge gaps and dispel misconceptions surrounding CRC.

## Teaser

High colorectal cancer awareness is associated with more positive attitudes toward colonoscopy in Palestine.

## INTRODUCTION

Colorectal cancer (CRC) is the third most prevalent cancer worldwide, with 1.9 million new cases and 930,000 deaths reported in 2020. Projections indicate a concerning trend of increasing incidence and mortality rates, with estimates reaching 3.2 million new cases and 1.6 million deaths by 2040.^1^ The escalating global prevalence of CRC also extends its impact to the Middle East, where inadequate awareness of the disease and its preventive measures underscores the urgent necessity for improved uptake of CRC screening. Data derived from the International Agency for Research on Cancer reveal that the Eastern Mediterranean Region is expected to register the second-highest increase in CRC-associated deaths between 2020 and 2040.^2^ Palestine, specifically the West Bank and the Gaza Strip, is no exception to this trend, as CRC represents the second most commonly diagnosed cancer after breast cancer with incidence rates of 15.3 and 10.2 per 100,000 general population, respectively.^3^

**CONTEXT**

**Key Objective**
Colorectal cancer (CRC) remains a public health concern in Palestine. Raising public awareness and influencing perceptions of CRC screening may augment the likelihood of early detection. Therefore, this national study aimed to assess CRC awareness and its influence on attitudes toward screening colonoscopy in Palestine.
**Knowledge Generated**
The study highlighted substantial knowledge gaps in CRC awareness in Palestinian adults, particularly pertaining to CRC signs/symptoms, risk factors, and causation myths. High awareness of CRC signs/symptoms and risk factors was associated with greater likelihood of demonstrating positive attitudes toward colonoscopy.
**Relevance**
Educational initiatives are needed to address knowledge gaps and dispel misconceptions surrounding CRC. Such initiatives may have the potential to lay the groundwork for implementing a screening program in Palestine and fostering more positive attitudes toward CRC screening and thus, resulting in early detection and intervention.

Approximately 70% of cancer-related mortalities occur within low- and middle-income countries, which could be primarily attributed to inadequate access to early diagnosis and treatment or delayed disease detection as a result of insufficient screening and awareness programs.^4^ The prognosis of CRC is notably more favorable when detected at an early stage compared with advanced and metastatic stages.^5^ Nevertheless, a substantial majority of patients receive their treatment at an advanced disease stage, resulting in a less favorable prognosis.^6^ Consequently, promoting a timely identification of CRC is of paramount importance, necessitating regular screening procedures (eg, colonoscopy).^7^ The American Cancer Society recommends individuals with an average risk of developing CRC to initiate regular screening at age 45 years, with individuals at higher risk commencing screening at an earlier age.^8^

Raising public awareness and influencing perceptions of CRC screening may augment the likelihood of early CRC detection. The introduction of screening programs has demonstrated a significant improvement in the survival rates and prognoses of patients with CRC.^9^ However, multiple studies conducted across various countries have consistently shown a widespread lack of awareness and knowledge concerning CRC screening.^10-14^ Although previous studies in Palestine have identified low levels of awareness concerning CRC signs/symptoms and risk factors,^15,16^ there is a noticeable gap in the literature when it comes to investigating the potential relationship between CRC awareness and individuals' attitudes toward colonoscopy as a screening procedure. Therefore, our national study aimed to assess the relationship between participants' awareness levels of CRC signs/symptoms, risk factors, and causation myths, and their attitudes toward colonoscopy. It also aimed to explore how a high level of awareness in each of these domains relates to agreement to certain aspects regarding colonoscopy.

## MATERIALS AND METHODS

### Study Design and Population

This nationwide cross-sectional study was conducted between July 2019 and March 2020. It targeted Palestinian adults age 18 years and older who were residing in the West Bank and Jerusalem, as well as the Gaza Strip. This age group accounts for approximately 62.2% of the total Palestinian population, which is estimated at around five million.^17^ Exclusion criteria encompassed individuals who were seeking care in oncology departments during the data collection period, those studying or working in the medical field, individuals holding citizenship from a country other than Palestine, and those who were unable to complete the questionnaire.

### Sampling Methods

In Palestine, health care services are provided through a network of health care facilities, including governmental institutions, nongovernmental organizations, private health care providers, and the United Nations Relief and Works Agency facilities. Government hospitals and primary health care centers offer services with minimal or no cost, making them the most accessible and commonly used health care providers for the majority of the population.^3^

Palestine is divided into 16 governorates, with 11 located in the West Bank and Jerusalem, and five in the Gaza Strip.^18^ To increase the potential of creating a comprehensive and representative study sample, we used a convenience sampling method to recruit participants from various settings.^15,16,19^ These settings included government hospitals, primary health care centers, as well as public spaces across 11 governorates in Palestine (seven in the West Bank and Jerusalem and four in the Gaza Strip). Public spaces included churches and mosques, shopping malls, local markets, parks, city centers, and public transportation stations. This approach was used to maximize the diversity and representativeness of the study cohort.^15,16,19^

### Data Collection and Measurement Tool

In this study, two validated assessment tools were adapted to collect data on public awareness regarding CRC signs/symptoms, risk factors, and causation myths: the Bowel Cancer Awareness Measure (BoCAM) and the Cancer Awareness Measure-Mythical Causes Scale (CAM-MYCS).^20,21^ Both questionnaires were developed by University College London and Cancer Research, United Kingdom. Modified versions of the BoCAM and CAM-MYCS were used after a thorough translation process, which involved converting the questionnaires from English to Arabic by two bilingual health care professionals. Subsequently, two other health care professionals independently back-translated them to English. All these professionals possessed proficiency in both languages and had experience in clinical research and survey design. Furthermore, the translated questionnaire underwent a rigorous evaluation by five independent experts in the fields of gastroenterology, coloproctology, and public health to ensure content validity and translation accuracy. To assess the clarity of the questions in the Arabic version of the questionnaire, a pilot study (n = 25) was conducted. The data from the pilot study were not included in the final analysis. The questionnaire's internal consistency was evaluated using Cronbach's alpha, which yielded an acceptable value of 0.81.

The questionnaire comprised five sections. The first section gathered sociodemographic information, including age, gender, educational level, employment status, monthly income, marital status, place of residence, presence of any chronic health conditions, knowing someone with cancer, and the site where the data were collected. The second section assessed participants' awareness of 12 different signs and symptoms associated with CRC. Respondents were asked to answer based on a 5-point Likert scale, ranging from 1 (strongly disagree) to 5 (strongly agree). In the third section, participants were queried about their awareness of 11 CRC risk factors, based on the same aforementioned 5-point Likert scale. Of the 12 signs and symptoms related to CRC, nine were retained from the original BoCAM,^20^ while additional three symptoms “feeling persistently full,” “unexplained loss of appetite,” and “unexplained generalized fatigue” were included based on other versions of the Cancer Awareness Measure.^22,23^ Similarly, 10 risk factors were adapted from the original BoCAM.^20^ A further risk factor corresponding to “smoking cigarettes” was added because of its high prevalence in the Palestinian population.^24^ The fourth section evaluated the participants' ability to identify 13 myths related to CRC causation to be incorrect. Among these 13 myths, 12 were derived from the original CAM-MYCS instrument.^20,21^ An additional myth regarding “eating burnt food” was added because of its popularity as a belief within the Palestinian community.^25^ The fifth section included 10 questions related to attitudes toward colonoscopy. These questions were adapted from previous studies^26-33^ and were based on the same aforementioned 5-point Likert scale.

Data were collected using Kobo Toolbox (Cambridge, MA), a secure and user-friendly tool for data collection that is accessible via smartphones.^34^ The data collection team had thorough training to effectively assist participants in completing the questionnaire through face-to-face interviews using Kobo Toolbox.

### Statistical Analysis

In accordance with the American Cancer Society guidelines, individuals at average risk for CRC should begin screening at age 45 years.^35^ To reflect this recommendation, study participants were divided into two age groups: 18-44 years and ≥45 years. Monthly income was also categorized into two groups: <1,450 New Israeli Shekel (NIS) and ≥1,450 NIS. This categorization aligns with Palestine's minimum wage standard of 1,450 NIS,^36^ which is approximately equivalent to $450 US dollars.

Descriptive statistics were used to summarize participant characteristics. Non-normally distributed continuous variables were summarized using median and IQR, and categorical variables were described using frequencies and percentages.

Prompt recognition of CRC signs/symptoms and risk factors was measured through questions based on a 5-point Likert scale, where strongly agree or agree represented correct responses, while strongly disagree, disagree, or not sure were considered incorrect answers. Myths surrounding the causation of CRC were also queried, where answers with disagree or strongly disagree were considered correct, and all other responses were considered incorrect.

Participants' awareness of CRC signs/symptoms, risk factors, and causation myths was evaluated using a scoring system adapted from previous studies.^15,16,19^ For each correctly recognized item, one point was given. The total awareness score of each domain was calculated and categorized into tertiles; the top tertile was considered high awareness, and the other two tertiles were considered low awareness. Similarly, participants were given one point for answering with agree or strongly agree on each of the questions related to attitudes toward colonoscopy. The total attitude score was calculated. The median attitude score was used to dichotomize the continuous overall attitude score; a score ≤5 was considered negative attitude and a score ≥6 was considered positive attitude.

Pearson's chi-square test was used to examine the association between displaying high awareness in each domain and agreeing on questions related to attitudes toward colonoscopy. This was followed by running a multivariable logistic regression to adjust for other covariates, including age, gender, education level, occupation, monthly income, marital status, place of residence, having a chronic disease, knowing someone with cancer, and site of data collection. This model was determined a priori based on previous studies.^15,16,19^ Similar analyses were also performed to examine the association between demonstrating high awareness in each domain and showing a positive attitude toward colonoscopy.

Missing data were hypothesized to be missed completely at random and thus, complete case analysis was used to handle them. Data were analyzed using Stata software version 17.0 (StataCorp, College Station, TX).

### Ethical Approval and Consent to Participate

Before data collection, ethical approval was obtained from the Research Ethics Committee at the Islamic University of Gaza, the Human Resources Development department at the Palestinian Ministry of Health, and the Helsinki Committee in the Gaza Strip. The study was conducted according to local guidelines and regulations. Participants received a comprehensive overview of the study objectives, with an emphasis on the voluntary nature of their participation. Participants provided written informed consent before their participation in the study.

## RESULTS

### Participant Characteristics

Of 5,254 individuals approached, 4,877 completed the questionnaire, yielding a response rate of 92.3%. A total of 4,623 questionnaires were included in the final analysis, with 210 excluded because of missing data and 44 for not meeting the inclusion criteria. Study participants had a median age (IQR) of 31.0 (24.0, 43.0) years, with 1,879 (40.6%) being males (Table 1).

**TABLE 1:** Characteristics of Study Participants

Characteristic	Total (N = 4,623)
Age, years, median (IQR)	31.0 (24.0-43.0)
Age group, years, No. (%)	
18-44	3,608 (78.1)
45 or older	1,015 (21.9)
Male gender, No. (%)	1,879 (40.6)
Educational level, No. (%)	
Secondary or below	2,217 (47.9)
Postsecondary	2,406 (52.1)
Occupation, No. (%)	
Unemployed/housewife	2,067 (44.7)
Employed	1,898 (41.1)
Retired	96 (2.1)
Student	562 (12.1)
Monthly income ≥1,450 NIS, No. (%)	3,039 (65.7)
Marital status, No. (%)	
Single	1,414 (30.5)
Married	3,067 (66.4)
Divorced/widowed	142 (3.1)
Residency, No. (%)	
Gaza Strip	1,923 (41.6)
West Bank and Jerusalem	2,700 (58.4)
Having a chronic disease, No. (%)	906 (19.6)
Knowing someone with cancer, No. (%)	2,395 (51.8)
Site of data collection, No. (%)	
Public spaces	1,450 (31.4)
Hospitals	1,659 (35.9)
Primary health care centers	1,514 (33.7)

Abbreviation: NIS, New Israeli Shekel.

### CRC Symptom Awareness and Attitudes Toward Colonoscopy

Overall, 1,849 study participants (40.0%) exhibited high awareness of CRC symptoms (Table 2). Participants with high CRC symptom awareness were more likely to agree on half of the questions related to colonoscopy, namely “willingness to pay for the procedure” (odds ratio [OR], 1.48 [95% CI, 1.26 to 1.74]), “being comfortable with a physician of a different gender performing the colonoscopy” (OR, 1.22 [95% CI, 1.07 to 1.39]), “perceiving that no other problems in life are more important than having a colonoscopy” (OR, 1.18 [95% CI, 1.04 to 1.35]), “reporting nice demeanor of physicians performing the colonoscopy” (OR, 1.20 [95% CI, 1.07 to 1.36]), and “believing that colonoscopy does not take too much time” (OR, 1.35 [95% CI, 1.19 to 1.52]).

**TABLE 2:** Summary of Association Between Demonstrating Good Awareness of Colorectal Cancer Symptoms and the Attitudes Toward Colonoscopy Among Study Participants

Question	Low Awareness (n = 2,774), No. (%)	High Awareness (n = 1,849), No. (%)	OR^a^ (95% CI)	*p*
If you had to pay for the colonoscopy, you would still do it	2,172 (78.3)	1,555 (84.1)	1.48 (1.26 to 1.74)	<.001
You would not feel ashamed to lie on a table to have a colonoscopy	1,679 (60.5)	1,186 (64.1)	1.13 (0.99 to 1.28)	.06
You would mind not if a physician with a gender different from yours performed the colonoscopy on you	1,822 (65.7)	1,307 (70.7)	1.22 (1.07 to 1.39)	.003
You would prefer a physician with a gender similar to yours to perform the colonoscopy on you	2,254 (81.3)	1,484 (80.3)	0.96 (0.82 to 1.12)	.58
You do not have other problems in your life more important than having a colonoscopy	1,837 (66.2)	1,302 (70.4)	1.18 (1.04 to 1.35)	.011
Having a colonoscopy is not too painful	678 (24.4)	511 (27.6)	1.13 (0.98 to 1.29)	.09
Physicians doing colonoscopy are nice to people	1,375 (49.6)	1,012 (54.7)	1.20 (1.07 to 1.36)	.003
Healthy people need to have a colonoscopy	1,367 (49.3)	928 (50.2)	1.00 (0.89 to 1.13)	.95
If you were destined to develop colorectal cancer, you would think that having a colonoscopy would have prevented it	1,007 (36.3)	639 (34.6)	0.92 (0.81 to 1.04)	.19
Having a colonoscopy does not take too much time	1,125 (40.6)	901 (48.7)	1.35 (1.19 to 1.52)	<.001

Abbreviation: OR, odds ratio.

^a^ Adjusted for age, gender, education level, employment status, monthly income, marital status, place of residence, presence of a chronic disease, knowing someone with cancer, and site of data collection.

The most reported agreement on questions related to colonoscopy was for “the willingness to pay for colonoscopy” in participants with high CRC symptom awareness (n = 1,555, 84.1%) and for “preferring a physician with a similar gender perform the colonoscopy” in those with low awareness (n = 2,254, 82.3%). Conversely, the least reported agreement in both participants with high (n = 511, 27.6%) or low (n = 678, 24.4%) CRC symptom awareness was for the belief that “having a colonoscopy is not too painful.”

### CRC Risk Factor Awareness and Attitudes Toward Colonoscopy

In total, 1,840 study participants (39.8%) demonstrated high awareness of CRC risk factors (Table 3). Notably, those with high awareness were more likely to express agreement to six of 10 questions related to colonoscopy. These questions were about “willingness to pay for the procedure” (OR, 1.46 [95% CI, 1.25 to 1.72]), “not feeling ashamed to have a colonoscopy” (OR, 1.14 [95% CI, 1.01 to 1.30]), “being comfortable with a physician of a different gender performing colonoscopy” (OR, 1.24 [95% CI, 1.09 to 1.41]), “believing that healthy people need to have a colonoscopy” (OR, 1.16 [95% CI, 1.02 to 1.30]), and “having a colonoscopy does not take too much time” (OR, 1.34 [95% CI, 1.19 to 1.51]).

**TABLE 3:** Summary of Association Between Demonstrating Good Awareness of Colorectal Cancer Risk Factors and the Attitudes Toward Colonoscopy Among Study Participants

Question	Low Awareness (n = 2,783), No. (%)	High Awareness (n = 1,840), No. (%)	OR^a^ (95% CI)	*p*
If you had to pay for the colonoscopy, you would still do it	2,200 (79.1)	1,527 (83.0)	1.46 (1.25 to 1.72)	<.001
You would not feel ashamed to lie on a table to have a colonoscopy	1,710 (61.4)	1,155 (62.8)	1.14 (1.01 to 1.30)	.039
You would mind not if a physician with a gender different from yours performed the colonoscopy on you	1,838 (66.0)	1,291 (70.2)	1.24 (1.09 to 1.41)	.001
You would prefer a physician with a gender similar to yours to perform the colonoscopy on you	2,206 (79.3)	1,532 (83.3)	1.25 (1.07 to 1.46)	.006
You do not have other problems in your life more important than having a colonoscopy	1,873 (67.3)	1,266 (68.8)	1.07 (0.94 to 1.21)	.33
Having a colonoscopy is not too painful	712 (25.6)	477 (25.9)	1.09 (0.95 to 1.26)	.20
Physicians doing colonoscopy are nice to people	1,418 (51.0)	969 (52.7)	1.11 (0.98 to 1.25)	.10
Healthy people need to have a colonoscopy	1,331 (47.8)	964 (52.4)	1.16 (1.02 to 1.30)	.018
If you were destined to develop colorectal cancer, you would think that having a colonoscopy would have prevented it	1,010 (36.3)	636 (34.6)	0.93 (0.82 to 1.06)	.30
Having a colonoscopy does not take too much time	1,141 (41.0)	885 (48.1)	1.34 (1.19 to 1.51)	<.001

Abbreviation: OR, odds ratio.

^a^ Adjusted for age, gender, education level, employment status, monthly income, marital status, place of residence, presence of a chronic disease, knowing someone with cancer, and site of data collection.

“Preference for a physician of the same gender to perform the colonoscopy” was the most frequently reported question to agree on among both participants with low (n = 2,206, 79.3%) or high awareness (n = 1,532, 83.3%) of CRC risk factors. By contrast, the least frequently reported agreement in both groups was to the belief that “colonoscopy is not too painful” (low-awareness group: n = 712, 25.6% *v* high-awareness group: n = 477, 25.9%).

### CRC Causation Myths Awareness and Attitudes Toward Colonoscopy

Only 219 participants (4.7%) demonstrated high awareness of CRC causation myths, while the majority (n = 4,404) did not (Table 4). Participants with high awareness of CRC causation myths were more likely to agree on three questions related to colonoscopy, specifically “the perception that having a colonoscopy is not too painful” (OR, 1.96 [95% CI, 1.48 to 2.62]), “the nice demeanor of physicians performing colonoscopy” (OR, 1.37 [95% CI, 1.03 to 1.82]), and “the belief that having a colonoscopy does not take too much time” (OR, 1.37 [95% CI, 1.04 to 1.80]). However, participants with high awareness of CRC causation myths were less likely to express a similar agreement to another three questions, namely “being comfortable with a physician of a different gender performing colonoscopy” (OR, 0.63 [95% CI, 0.47 to 0.83]), “believing that no other problems in life are more important than having a colonoscopy” (OR, 0.72 [95% CI, 0.54 to 0.96]), and “thinking that healthy people need to have a colonoscopy” (OR, 0.71 [95% CI, 0.53 to 0.94]).

**TABLE 4:** Summary of Association Between Demonstrating Good Awareness of Colorectal Cancer Causation Myths and the Attitudes Toward Colonoscopy Among Study Participants

Question	Low Awareness (n = 4,404), No. (%)	High Awareness (n = 219), No. (%)	OR^a^ (95% CI)	*p*
If you had to pay for the colonoscopy, you would still do it	3,556 (80.7)	171 (78.1)	0.82 (0.58 to 1.15)	.24
You would not feel ashamed to lie on a table to have a colonoscopy	2,731 (62.0)	134 (61.2)	0.81 (0.60 to 1.07)	.14
You would mind not if a physician with a gender different from yours performed the colonoscopy on you	2,999 (68.1)	130 (59.4)	0.63 (0.47 to 0.83)	.001
You would prefer a physician with a gender similar to yours to perform the colonoscopy on you	3,559 (80.8)	179 (81.7)	1.10 (0.77 to 1.58)	.61
You do not have other problems in your life more important than having a colonoscopy	3,005 (68.2)	134 (61.2)	0.72 (0.54 to 0.96)	.025
Having a colonoscopy is not too painful	1,094 (24.8)	95 (43.4)	1.96 (1.48 to 2.62)	<.001
Physicians doing colonoscopy are nice to people	2,254 (51.2)	133 (60.7)	1.37 (1.03 to 1.82)	.029
Healthy people need to have a colonoscopy	2,206 (50.1)	89 (40.6)	0.71 (0.53 to 0.94)	.016
If you were destined to develop colorectal cancer, you would think that having a colonoscopy would have prevented it	1,569 (35.6)	77 (35.2)	0.95 (0.71 to 1.27)	.75
Having a colonoscopy does not take too much time	1,914 (43.5)	112 (51.1)	1.37 (1.04 to 1.80)	.027

Abbreviation: OR, odds ratio.

^a^ Adjusted for age, gender, education level, employment status, monthly income, marital status, place of residence, presence of a chronic disease, knowing someone with cancer, and site of data collection.

“Preference for a physician with a similar gender to perform the colonoscopy” was the most frequently reported question to agree on among participants with low (n = 3,559, 80.8%) or high (n = 179, 81.7%) awareness. Conversely, the least frequently reported agreement among participants with low awareness of CRC causation myths was for “the perception that having a colonoscopy is not too painful” (n = 1,094, 24.8%), while it was for the belief that “colonoscopy could prevent CRC if they were destined to have it” (n = 77, 35.2%) among those with high awareness.

### Association Between High CRC Awareness and Positive Attitudes Toward Colonoscopy

Participants with high CRC symptom awareness were more likely than those with low awareness to display positive attitude toward colonoscopy (OR, 1.21 [95% CI, 1.07 to 1.37]; Table 5). Similarly, participants with high CRC risk factor awareness had a greater likelihood than those with low awareness to display positive attitude toward colonoscopy (OR, 1.20 [95% CI, 1.07 to 1.36]). There was no association between exhibiting a high awareness of CRC causation myths and showing a positive attitude toward colonoscopy.

**TABLE 5:** Association of Demonstrating High Awareness in Each Domain With Showing Positive Attitude Toward Colonoscopy

Domain	Negative Attitude (n = 2,083), No. (%)	Positive Attitude (n = 2,540), No. (%)	OR^a^ (95% CI)	*p*
Awareness of CRC signs/symptoms				
Low	1,313 (63.0)	1,461 (57.5)	Ref	Ref
High	770 (37.0)	1,079 (42.5)	1.21 (1.07 to 1.37)	.002
Awareness of CRC risk factors				
Low	1,292 (62.0)	1,491 (58.7)	Ref	Ref
High	791 (38.0)	1,049 (41.3)	1.20 (1.07 to 1.36)	.003
Awareness of CRC causation myths				
Low	1,984 (95.2)	2,420 (95.3)	Ref	Ref
High	99 (4.8)	120 (4.7)	0.92 (0.70 to 1.22)	.56

Abbreviations: CRC, colorectal cancer; OR, odds ratio; Ref, reference.

^a^ Adjusted for age, gender, education level, employment status, monthly income, marital status, place of residence, presence of a chronic disease, knowing someone with cancer, and site of data collection.

## DISCUSSION

This study investigated the association between demonstrating high awareness of CRC symptoms, risk factors, and causation myths and manifesting a positive attitude toward colonoscopy. Overall, the results revealed that participants with high awareness of CRC symptoms were more likely to display a positive attitude toward colonoscopy. Likewise, individuals with high awareness of CRC risk factors showed a greater propensity for a positive attitude toward colonoscopy compared with those with low awareness. However, there was no association between exhibiting a high level of awareness of CRC causation myths and displaying a positive attitude toward colonoscopy. These results may provide important information for facilitating the development of a CRC screening program in Palestine and further public policymaking toward prevention and early diagnosis of CRC.

Only 40% of our study participants exhibited high awareness of CRC symptoms, consistent with findings from previous studies.^15^ Additionally, participants with high awareness of CRC symptoms were more likely to express a positive attitude toward colonoscopy. Most participants with high CRC symptom awareness had a “willingness to pay for the procedure,” with 84.1% expressing readiness to cover the costs, significantly higher than participants with low CRC symptom awareness (78.3%). Similarly, a study in Guangzhou revealed that 91.7% of participants expressed a willingness to pay for CRC screening.^37^ This indicates that increasing CRC awareness can help improve positive attitude toward CRC screening. As individuals' knowledge grows, so does their favorable disposition toward colonoscopy. A study involving Jordanian adults revealed that health education interventions positively affected participants' understanding and attitudes regarding CRC screening.^38^ In addition, a previous systematic review highlighted that a lack of knowledge of GI symptoms was a barrier to implementing CRC screening in Asia.^39^ This emphasizes the significant influence of public understanding of CRC screening on individual attitudes, indicating that as knowledge increases, so does the receptiveness to colonoscopy.

Overall, 39.8% of participants demonstrated high awareness of CRC risk factors, aligning with previously published studies.^16^ Significantly, those with increased awareness were more likely to exhibit a positive attitude toward colonoscopy compared with their counterparts with lower awareness. The most frequently observed agreement across participants, regardless of their awareness level of CRC risk factors, was for the “preference for a physician of the same gender to perform the colonoscopy.” Multiple studies have previously indicated a significant influence of gender preferences on the likelihood of pursuing endoscopic evaluations, particularly among women.^40-43^ Interestingly, a study in China reported that women were more inclined than men to participate in a CRC screening program.^29^ Hence, cultural factors, including values associated with religion and spirituality, are linked to various cancer prevention and control behaviors.^44^ Therefore, efforts aimed at enhancing CRC screening participation should incorporate gender-specific strategies to tackle psychological barriers. This could be achieved through targeted educational interventions and proactive encouragement by health care professionals to undergo CRC screening.

Furthermore, the effectiveness of screening initiatives relies on public participation, which, in turn, is influenced by the public's understanding of its importance. Previous research has indicated that awareness of CRC signs and symptoms significantly influences screening participation.^33^ Additionally, an understanding of CRC risk factors independently predicts individuals' intentions to participate in screening.^33,45^

Interestingly, the least frequently observed agreement across participants, regardless of their level of awareness, was for the belief that “colonoscopy is not too painful.” McLachlan et al systematically reviewed 56 studies to delineate patients' experiences with screening colonoscopy. The authors identified significant barriers, such as concerns about bowel preparation, anxiety, anticipation of pain, insufficient knowledge, and fear of cancer, as obstacles to screening colonoscopy.^46^ Additionally, a study of adults in the Saudi population discovered that the fear of painful procedures emerged as a prominent barrier,^47^ aligning with similar regional and global studies.^48-50^ Thus, to overcome these barriers, counseling sessions should be arranged to enhance patients' comfort levels and address their concerns regarding the procedure.

Only 5% of participants in our study demonstrated high awareness of CRC causation myths. Notably, there was no association between exhibiting a high awareness of CRC causation myths and showing a positive attitude toward colonoscopy. The observed deficiency in CRC causation myth awareness suggests a gap in public health education. This highlights the importance of disseminating accurate information to dispel prevalent misconceptions. Mythical beliefs may contribute to increased anxiety, particularly when the perceived risk is beyond individual control.^51^

Enhancing participation in screening programs for CRC necessitates raising awareness of its signs/symptoms, risk factors, and dispelling common misconceptions regarding its causation. Comprehensive educational campaigns are vital for fostering a nuanced understanding of CRC attributes, particularly among individuals with low awareness. Such efforts play a pivotal role in shaping attitudes toward CRC screening. Addressing misconceptions is anticipated to mitigate the fear and stigma associated with CRC; therefore, health care systems and primary care physicians should actively incorporate CRC awareness as a mandatory component in patient interactions. This integration seeks to mitigate identified barriers linked to fear and perception of pain.

The major strengths of this study include the large sample size drawn from diverse regions across Palestine and the high response rate. Additionally, the use of validated tools, BoCAM and CAM-MYCS, enhances the reliability of the collected data. Moreover, face-to-face interviews during data collection minimized the likelihood of participants using online sources for accurate responses.

However, the study has some limitations. The use of convenience sampling might not fully create a representative sample of the Palestinian population, limiting the generalizability of the findings. Nonetheless, the large sample size, high response rate, and diverse regional data collection help mitigate this limitation. In addition, the exclusion of individuals from oncology departments and those with medical backgrounds may have potentially resulted in a decreased number of participants with presumed good CRC awareness. However, this was done to maximize the potential to measure the public awareness of CRC. Finally, the study assessed participants' perceived knowledge and did not evaluate the awareness of individuals with actual CRC symptoms.

In conclusion, this study highlighted substantial knowledge gaps in CRC awareness in Palestinian adults, particularly pertaining to CRC signs/symptoms, risk factors, and causation myths. Interestingly, while being well informed about CRC symptoms and risk factors was linked to a positive attitude toward colonoscopy, the same association was not observed with high awareness of CRC causation myths. This emphasizes the need for tailored public health campaigns designed to dispel existing misconceptions and promote CRC screening. Such initiatives have the potential to lay the groundwork for implementing a screening program in Palestine and fostering more positive attitudes toward CRC screening and thus resulting in early detection and intervention.
